# Regulation of AMPK Activity by CRBN Is Independent of the Thalidomide-CRL4^CRBN^ Protein Degradation Axis

**DOI:** 10.3390/ph14060512

**Published:** 2021-05-26

**Authors:** Seung-Joo Yang, Seungje Jeon, Jeong Won Baek, Kwang Min Lee, Chul-Seung Park

**Affiliations:** 1 School of Life Sciences and Cell Logistics Research Center, Gwangju Institute of Science and Technology (GIST), Gwangju 61005, Korea; ysj@gist.ac.kr (S.-J.Y.); jeonsj@gist.ac.kr (S.J.); baekjw0528@gist.ac.kr (J.W.B.); 2 Department of Life Science and Environmental Biochemistry, Life and Industry Convergence Research Institute, Pusan National University, Miryang 50463, Korea

**Keywords:** thalidomide, AMP-activated protein kinase, cereblon

## Abstract

Cereblon (CRBN), a primary target of immune-modulatory imide drugs (IMiDs), functions as a substrate receptor in the CUL4-RBX1-DDB1-CRBN (known as CRL4^CRBN^) E3 ubiquitin ligase complex. Binding of IMiDs to CRBN redirects the CRL4^CRBN^ E3 ubiquitin ligase to recruit or displace its substrates. Interaction between CRBN and the AMPK α subunit leads to CRL4^CRBN^-dependent degradation of the γ subunit and inhibits AMPK activity. However, the effect of thalidomide on the function of CRBN as a negative regulator of AMPK through interaction with the α subunit remains unclear. Here, we show that thalidomide does not affect AMPK activation or the binding affinity between CRBN and the AMPK α subunit. Thalidomide had no effect on AMPK activity independent of CRBN expression. The N-terminal region and C-terminal tail of CRBN, which is distinct from the IMiD binding site, were critical for interaction with the AMPK α subunit. The present results suggest that CRL4^CRBN^ negatively regulates AMPK through a pathway independent from the CRBN-IMiD binding region.

## 1. Introduction

Thalidomide was widely prescribed as a sedative-hypnotic for pregnant women in the late 1950s and early 1960s to relieve morning sickness, but was withdrawn from the market because of its teratogenic effects [1,2]. However, further studies demonstrated the therapeutic potential of thalidomide. Thalidomide and its derivatives are now widely used for the treatment of hematologic malignancies, such as multiple myeloma [3,4] and 5q deletion-associated myelodysplastic syndrome (MDS) [5].

Cereblon (CRBN) is a primary target of immune-modulatory imide drugs (IMiDs), such as thalidomide [6] and its derivatives lenalidomide and pomalidomide [7,8]. CRBN functions as a substrate receptor for the Cullin-4A/B RING E3 ubiquitin ligase (CRL4) complex composed of Cullin-4A/B, damage-specific DNA-binding protein 1 (DDB1), and RING-box protein 1 (RBX1), and is responsible for the recruitment of substrates for degradation by the ubiquitin-proteasome pathway [6,9]. CRL4^CRBN^ mediates ubiquitination of endogenous substrates, such as Ca^2+^- and voltage-activated K^+^ (BK) channels [10,11], Meis homeobox 2 (MEIS2) [12], the CLC-1 chloride channel [13,14], and glutamine synthetase (GS) [15]. Binding of IMiDs to CRBN alters the substrate specificity of CRL4^CRBN^. IMiDs block the interaction between endogenous substrates and CRBN, thereby inhibiting their ubiquitination and degradation [12]. However, IMiD binding to CRBN promotes the recruitment of neo-substrates, such as Ikaros (IKZF1), Aiolos (IKZF3) [16,17], casein kinase 1A1 (CK1α) [18], Sall4 [19], and p63 [20], leading to their ubiquitin-dependent degradation. CRBN consists of an amino-terminal domain (NTD), a helical bundle domain (HBD) that contains the DDB1 binding region, and a carboxy-terminal domain (CTD). The CTD includes the IMiD binding pocket, which is composed of three tryptophan residues (Trp380, Trp386, and Trp400) and a phenylalanine residue (Phe402). These residues form a hydrophobic pocket that accommodates the glutarimide moiety of IMiDs. By contrast, the phthalimide rings are exposed to the solvent and change the molecular surface of CRBN, thereby modulating its substrate specificity [12,21].

AMPK, the master regulator of energy homeostasis, is a heterotrimer complex composed of a catalytic α subunit and two regulatory subunits, β and γ [22,23]. The catalytic α subunit contains a serine/threonine kinase domain at the N-terminus that includes threonine 172, which is phosphorylated by upstream kinases, such as liver kinase B1 and Ca^2+/^calmodulin-dependent protein kinase β [24,25]. The β subunit acts as a scaffold for the α and γ subunits, and the carbohydrate-binding module (CBM) on the β subunit allows AMPK to detect glycogens [26]. Moreover, the serine 108 (Ser108) within CBM was reported as a cis-autophosphorylation site. The small compound drug, A769662, allosterically activates AMPK, and when combined with allosteric activation by AMP it can, in certain instances, completely activate AMPK in the absence of α-Thr172 phosphorylation. [27]. The γ subunit contains four cystathionine β-synthase sites. The two interfaces between CBS1 and CBS2, as well as between CBS3 and CBS4, form each two clefts with the potential to bind two adenine nucleotides. AMPK is activated in response to energy stress by sensing increases in the AMP:ATP and AMP:ADP ratios; activated AMPK thus inhibits ATP-consuming anabolic pathways and promotes ATP-generating catabolic pathways to restore energy balance [22,28,29]. 

In previous work from our group, we showed that CRL4^CRBN^-dependent degradation of the AMPK γ subunit inhibited AMPK activity [30,31]. However, the effect of thalidomide on the CRBN-mediated regulation of AMPK has not been reported to date. Here, we show that thalidomide had no effect on AMPK activity or on the interaction between CRBN and AMPK. Its activity was inhibited by wild-type (WT) CRBN and a thalidomide-resistant mutant in the presence or absence of thalidomide. We showed that both the N- and C-terminal domains of CRBN mediated the interaction with the AMPK α subunit. In particular, the binding region in the CTD did not overlap with the IMiD binding pocket. Taken together, the results demonstrated that the inhibitory action of CRBN against AMPK activity was independent of the presence of thalidomide.

## 2. Results

### 2.1. Thalidomide Does Not Affect AMPK Activity or the Interaction between CRBN and the AMPK α Subunit

**Figure 1:**
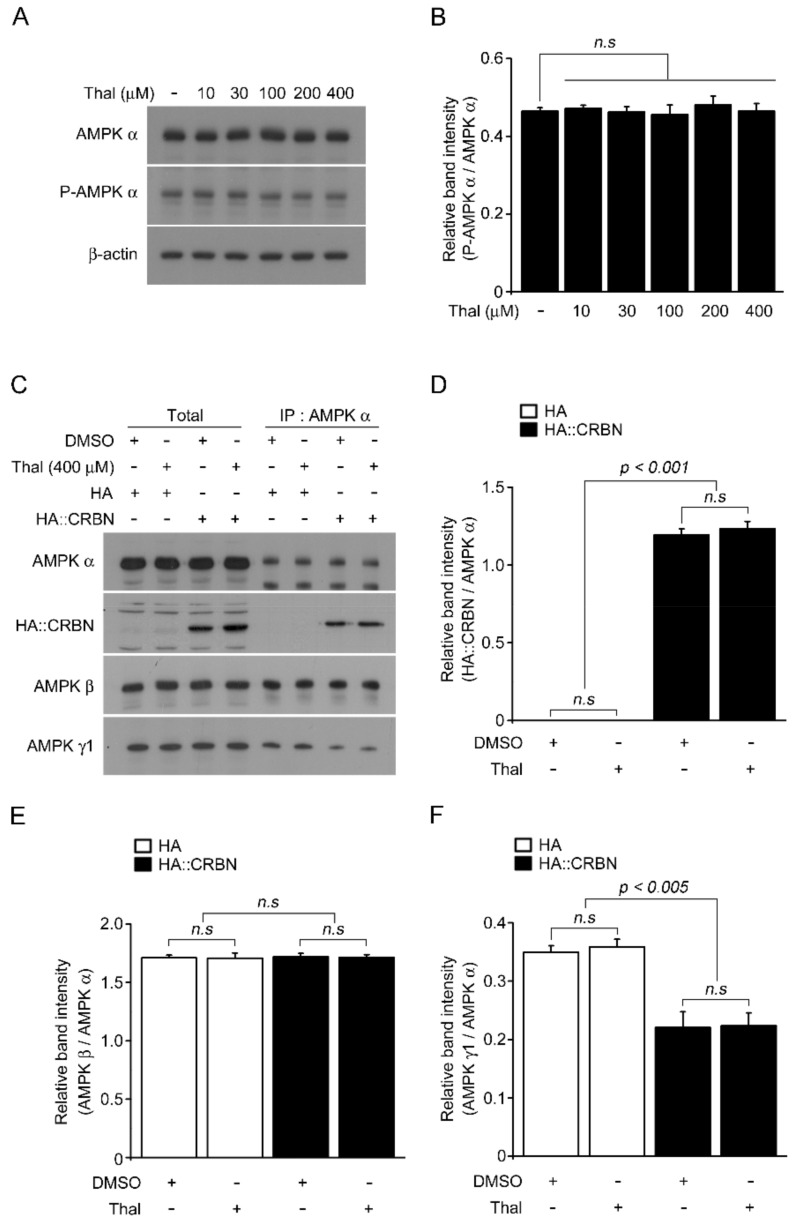
Effect of thalidomide on AMPK activity and binding affinity of CRBN to AMPK. (**A**) HepG2 cells were treated with different concentrations of thalidomide (0, 10, 30, 100, 200, and 400 μM) for 24 h. Cell lysates were subjected to Western blot analysis with anti-AMPK α, anti-p-AMPK α, and β-actin antibodies. β-actin was used as a loading control. (**B**) The band intensity of p-AMPK α in (**A**) was quantified by densitometry and normalized to total AMPK α. (**C**) HepG2 cells were transiently transfected with empty HA or HA:CRBN. After 24 h, the cells were treated with or without thalidomide (400 μM) for 24 h. Cell lysates were immunoprecipitated with anti-AMPK α antibody and probed with anti-AMPK α, anti-HA, anti-AMPK β, and anti-AMPK γ antibodies. (**D**–**F**) Relative band intensities were measured by densitometric analysis of the blot in (**C**). Thal indicates thalidomide. Error bars represent the SEM (*n* = 4). The results shown were obtained from four independent experiments.

We previously demonstrated that CRBN inhibits the activation of AMPK by interacting directly with the α subunit of AMPK [30]. Because thalidomide alters the substrate specificity of CRBN, we examined whether thalidomide had an effect on CRBN-dependent regulation of AMPK activity. First, we measured the phosphorylation of AMPK in the human liver cancer cell line HepG2 after treatment with various doses of thalidomide for 24 h. The results showed that thalidomide had no effect on total and p-AMPK α levels (Figure 1A,B). To determine whether thalidomide affected the binding affinity of the AMPK α subunit to CRBN, their interaction was validated by co-immunoprecipitation in the presence or absence of thalidomide. Cells were transiently transfected with HA-tagged CRBN, which was immunoprecipitated with endogenous AMPK α subunit (Figure 1C). There was no difference in the binding affinity of the α subunit to CRBN between DMSO and thalidomide-treated cells (Figure 1D). The amount of γ subunit in the precipitated complex was reduced by exogenous CRBN, as previously reported, and was not affected by thalidomide (Figure 1F). 

### 2.2. Thalidomide Does Not Alter the Inhibitory Effect of CRBN on AMPK

**Figure 2:**
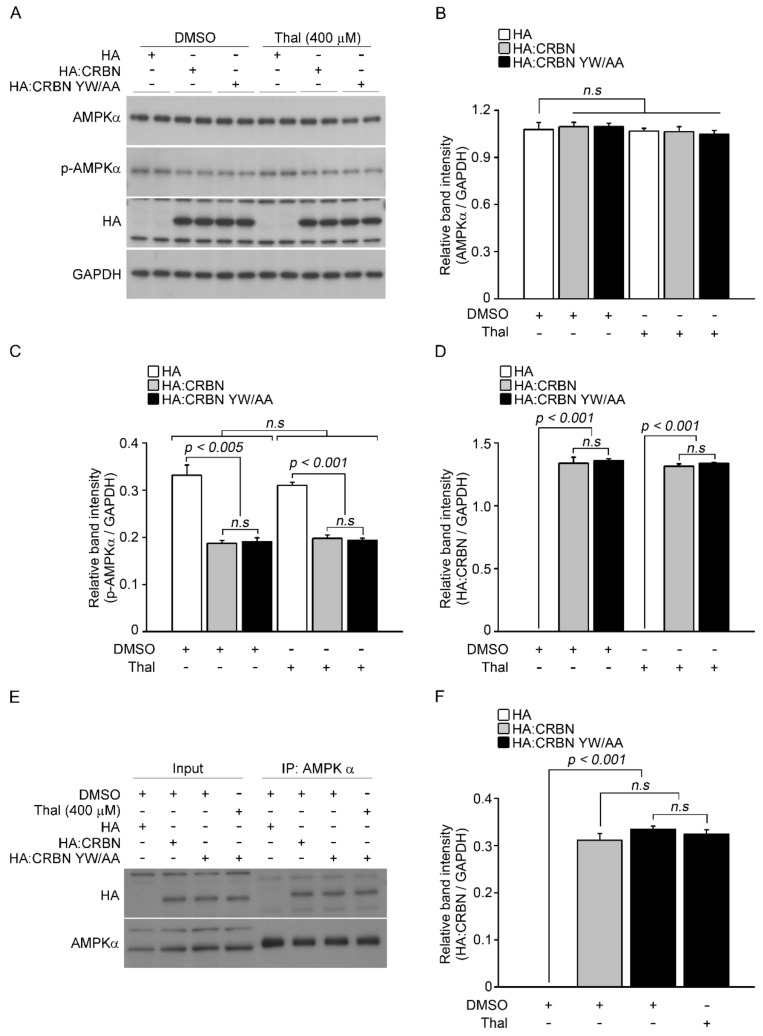
Regulation of AMPK activity by exogenous CRBN in the presence of thalidomide. (**A**) HepG2 cells were transiently transfected with empty HA, HA:CRBN, or HA:CRBN YW/AA. After 24 h, cells were treated with or without thalidomide (400 μM) for 24 h. Cell lysates were analyzed by Western blotting with the indicated antibodies. GAPDH was used as a loading control. (**B**–**D**) Relative band intensities were determined by densitometric analysis of the blot shown in (**A**). (**E**) Cell lysates were prepared from HepG2 cells transfected with empty HA, HA:CRBN, or HA:CRBN YW/AA in the presence or absence of thalidomide. Proteins were immunoprecipitated with an anti-AMPK α antibody and analyzed by Western blotting with anti-HA and anti-AMPK α antibodies. (**F**) Relative band intensities were measured by densitometric analysis of the blot in (**E**). Error bars represent the SEM (*n* = 4). The results shown were obtained from four independent experiments.

The effect of thalidomide on the inhibition of AMPK by CRBN was examined by assessing *p*-AMPK α levels in response to ectopic expression of CRBN in the presence of thalidomide (Figure 2A). The thalidomide binding-deficient mutant CRBN YW/AA (mutation of Tyr384 and Trp386 to Ala) [8] was used to further examine the effect of thalidomide. The levels of p-AMPK α decreased to similar levels in CRBN WT and CRBN YW/AA transfected cells, and exposure to thalidomide had no effect on the inhibition of AMPK by CRBN (Figure 2C). To confirm that thalidomide did not affect the interaction between CRBN and the α subunit, we examined the interaction between AMPK α and the CRBN YW/AA mutant by transiently transfecting HepG2 cells with HA-tagged CRBN WT or YW/AA mutant, followed by immunoprecipitation with an AMPK α antibody. The CRBN YW/AA mutant bound with similar affinity to the α subunit as CRBN WT in the presence and absence of thalidomide, suggesting that CRBN inhibits AMPK activity following interaction with its α subunit, regardless of thalidomide binding (Figure 2E,F).

### 2.3. CRBN Knockout-Induced Increase in AMPK Activity Is Not Affected by Thalidomide

**Figure 3:**
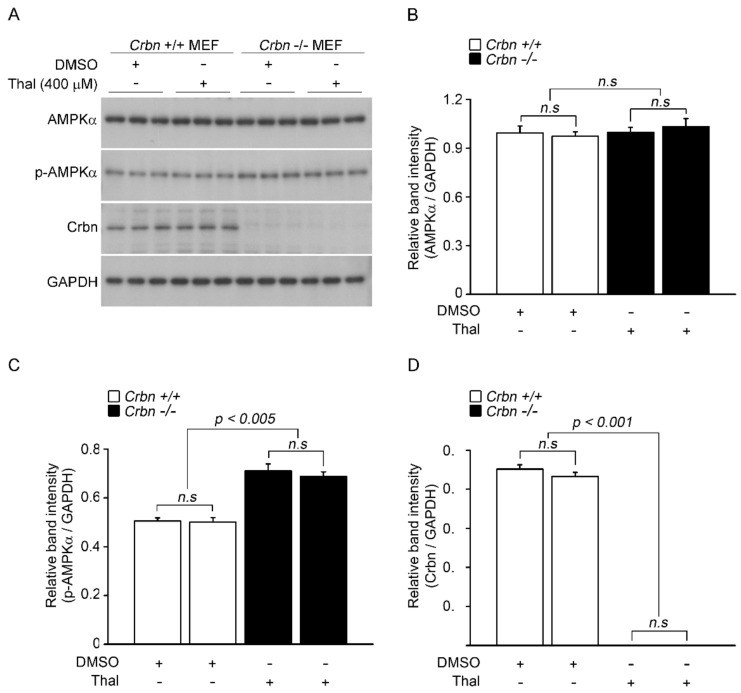
Action of thalidomide on AMPK activity in the absence of CRBN. (**A**) Wild-type and Crbn-/- MEFs were treated with thalidomide (400 μM) for 24 h. Cell lysates were analyzed by Western blotting with anti-AMPK α, anti-p-AMPK α, anti-CRBN, and anti-GAPDH. GAPDH was used as a loading control. (**B**–**D**) Relative band intensities were measured by densitometric analysis of the blot in (**A**). Error bars represent the SEM (*n* = 4). The results shown were obtained from four independent experiments.

**Figure 4:**
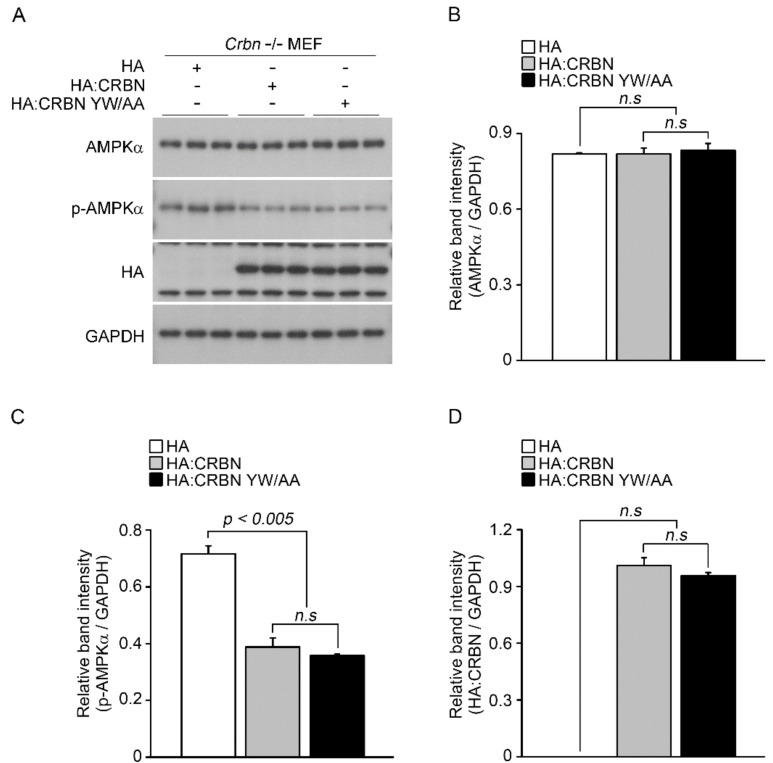
Effect of exogenous CRBN on AMPK activity in the absence of CRBN. (**A**) *Crbn*^-/-^ MEFs were transiently transfected with empty HA, HA:CRBN, or HA:CRBN YW/AA. After 24 h, cells were treated with or without thalidomide (400 μM) for 24 h. Cell lysates were subjected to Western blot analysis with anti-AMPK α, anti-p-AMPK α, anti-HA, and anti-GAPDH antibodies. GAPDH was used as a loading control. (**B**–**D**) Relative band intensities were determined by densitometric analysis of the blot shown in (**A**). Error bars represent the SEM (*n* = 3). The results shown were obtained from three independent experiments.

Because CRBN deficiency in mice increases the phosphorylation of AMPK [32], we examined the effect of thalidomide on AMPK activity in the absence of CRBN. To this end, *p*-AMPK α levels were measured following treatment with DMSO or thalidomide in wild-type and *Crbn*^-/-^ MEFs (Figure 3A). As reported previously, depletion of CRBN increased the phosphorylation, but not the total levels, of the α subunit of AMPK (Figure 3B,C). No significant differences in the levels of p-AMPK α were observed between DMSO- and thalidomide-treated WT MEFs. Moreover, thalidomide treatment did not alter the increased p-AMPK α levels in *Crbn*^-/-^ MEFs (Figure 3C). To further examine the effect of a thalidomide binding-deficient mutant on AMPK activity in *Crbn*^-/-^ MEFs, cells were transiently transfected with WT CRBN or CRBN YW/AA (Figure 4A). In the absence of CRBN, the inhibitory effect of CRBN YW/AA on AMPK activity was comparable with that of WT CRBN (Figure 4C). These data suggest that the effect of thalidomide on AMPK activation is not affected by the level of CRBN.

### 2.4. The AMPK α Subunit Binds to the N and C-Termini of CRBN

**Figure 5:**
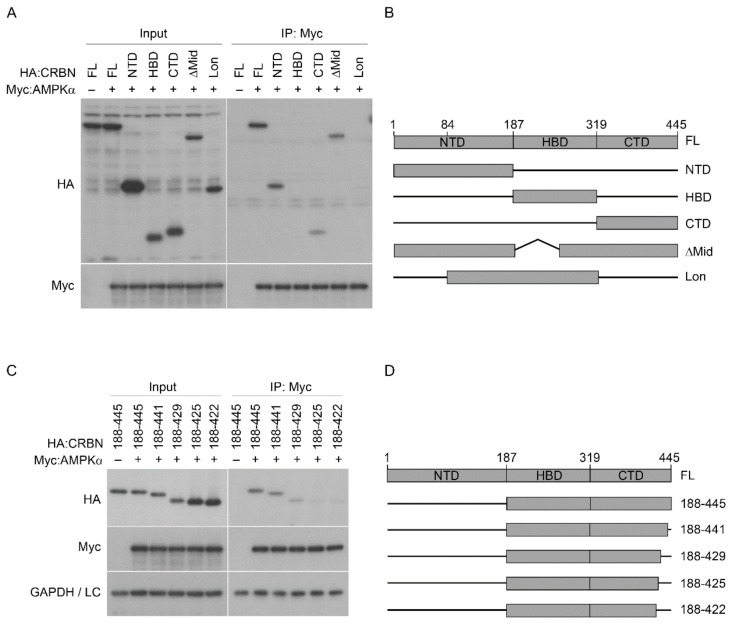
AMPK α subunit interacts with two distinct regions of CRBN, the N-terminal and C-terminal domains. (**A**) SH-SY5Y cells were transiently co-transfected with HA-tagged domain mutants of CRBN and Myc:AMPK α or empty Myc. Cell lysates were immunoprecipitated with anti-Myc antibody, and the precipitated and input fractions were analyzed by Western blotting with anti-HA and anti-Myc antibodies. (**B**) Schematic diagrams of full-length CRBN and the domain mutants used in (**A**). The numbers represent the amino acids where each construct starts and ends. (**C**) Cell lysates from SH-SY5Y cells transfected with HA-tagged serial deletion mutants of CRBN and Myc:AMPK α or empty Myc were subjected to immunoprecipitation with anti-Myc antibody followed by Western blotting with the indicated antibodies. (**D**) Schematic diagram of the full-length CRBN and serial deletion mutants used in (**C**). LC indicates the IgG light chain. The results shown were obtained from three independent experiments.

CRBN interacts with the AMPK α subunit via separate binding sites in the N-terminal (1–100) and C-terminal (393–422) regions [30]; however, the CRBN domain that interacts with the α subunit has not been identified. To identify the binding region, we generated a series of deletion mutants of the NTD, HBD, CTD, a DDB1-binding defective mutant (∆Mid), and a Lon domain (Lon) mutant. We then performed immunoprecipitation assays with anti-Myc antibody after co-transfection of SH-SY5Y cell lines with Myc-tagged AMPK α subunit and HA-tagged CRBN mutants (Figure 5A,B). The NTD, CTD, Lon, and ∆Mid mutants, but not the HBD mutant, robustly co-precipitated with the α subunit, suggesting that the N- and C-terminal regions of CRBN are involved in the interaction with the α subunit (Figure 5A). The pathogenic CRBN mutant lacking the last 24 amino acids bound to the α subunit with lower affinity than WT CRBN [33], suggesting that the deleted region is responsible for the interaction between the α subunit of AMPK and CRBN. To narrow down the region, we constructed a deletion mutant (188–445) that did not include the NTD, and we serially deleted the amino acid residues in the C-terminal tail region of the 188–445 mutant (Figure 5D). The interaction between the α subunit and the deletion mutants was then tested by immunoprecipitation. There were no significant differences in binding affinity for the α subunit between the 188–445 and 188–441 mutants. The 188–429 mutant interacted with the α subunit with lower affinity, and the 188–425 mutant completely lost binding affinity for the α subunit (Figure 5C). Taken together, these results suggest that the α subunit interacts with two different regions of CRBN, the N-terminal region and the amino acids 426–440 in the C-terminal tail.

## 3. Discussion

AMPK is a highly conserved master regulator of cellular energy metabolism that can be activated by increases in the AMP:ATP or ADP:ATP ratio [22,23]. AMPK is implicated in the regulation of hepatic glucose and lipid metabolism, thereby affecting the energy status of the whole body [34,35]. Moreover, AMPK is considered to be a major pharmacological target protein for the treatment of metabolic diseases [36,37,38]. We previously showed that CRBN interacts with the AMPK α subunit and inhibits the activation of AMPK in vitro and in vivo [30,33]. We demonstrated that inhibition of AMPK activity by CRBN is mediated by CRL4^CRBN^-dependent degradation of the AMPK γ subunit following interaction between CRBN and the AMPK α subunit [30,31,32]. CRBN, a key protein involved in autosomal recessive nonsyndromic mental retardation [39], is the direct target of IMiDs, including thalidomide and its derivatives [6,7,8]. The binding of neo-substrates, such as IKZF1, IKZ3, and CK1α to CRBN and their ubiquitination, is induced by IMiDs. Increased ubiquitination and proteasomal degradation of these proteins induced by IMiDs is the molecular mechanism underlying the treatment of multiple diseases, such as multiple myeloma [16,17] and MDS with deletion of chromosome 5q [18]. Moreover, thalidomide causes teratogenic effects by promoting the accumulation of MEIS2 [12] and the degradation of neo-substrates, such as SALL4 [19], ∆Np63, and Tap63 [20]. Although various substrates of CRBN are affected by IMiDs, the effect of thalidomide on CRBN-dependent regulation of AMPK is not fully understood. 

Here we show, for the first time, that CRBN functions as a negative regulator of AMPK in a thalidomide-independent manner, as determined by the identification of different binding regions to AMPK and thalidomide. Thalidomide did not affect the levels of p-AMPK α or the interaction between the α subunit and CRBN in HepG2 cells. Consistently, thalidomide did not alter the levels of the γ subunit in the AMPK complex (Figure 1). Ectopic expression of CRBN inhibited the phosphorylation of the α subunit independent of the presence or absence of thalidomide (Figure 2 and 4). Moreover, treatment of *Crbn*^-/-^ MEFs with thalidomide did not alter the levels of p-AMPK α (Figure 3), suggesting that the regulation of α-Thr172 phosphorylation-dependent AMPK activation by thalidomide is independent from the expression levels of CRBN. The binding affinity of endogenous substrates, such as MEIS2 [12] and GS [15], to CRBN is altered in the presence of IMiDs because their binding region overlaps with, or is adjacent to, the IMiD pocket in the CTD of CRBN. Here, immunoprecipitation assays were used to show that the α subunit interacts with CRBN through separate binding sites in the N-terminal (1–187) and C–terminal (426-440) regions (Figure 5). These results indicate that thalidomide does not affect the regulation of AMPK by CRBN because the interaction module for protein binding is distinct from the IMiD-binding cage. 

In our previous study, we showed that depletion of the *Crbn* gene improves the symptoms of metabolic syndrome by activating AMPK [34]. This led us to hypothesize that CRBN could be a potential therapeutic target for the treatment of metabolic disorders. Chemicals that inhibit the interaction between CRBN and AMPK could be utilized as anti-diabetic drugs, either alone or in combination with conventional therapies. The present findings of the role of CRBN on the effect of thalidomide on α-Thr172 phosphorylation-dependent AMPK activation improve our understanding of the physiological function of CRBN at the molecular level under normal and diseased conditions. This improved understanding of the molecular mechanisms of CRBN function may lead to the development of novel therapeutic approaches for the treatment of human diseases.

## 4. Materials and Methods

### 4.1. Plasmid Construction and Transfection

Wild-type and mutant CRBN were subcloned from the pcDNA3.0-HA/CRBN plasmid obtained previously and inserted into the pEBB-3HA vector. Construction of a plasmid encoding Myc tagged rat Ampk (Myc:AMPK α) was described previously. Cells were seeded 1 day before transfection at 70% confluency in 6-well plates and then transfected with 2 μg of plasmid per well using Lipofectamine 2000 (Invitrogen), according to the manufacturer’s protocols.

### 4.2. Cell Culture

HepG2 cells (human liver cancer cell line), mouse embryonic fibroblasts (MEFs), and SH-SY5Y cells (human neuroblastoma cell line) were cultured in Dulbecco’s modified Eagle’s medium (Hyclone) and supplemented with 10% (*v/v*) fetal bovine serum (Hyclone). WT and *Crbn^-/-^* MEFs were isolated from E14.5 embryos born to heterozygous intercrosses and assayed at passages 3–6, as previously described [40].

### 4.3. Western Blot Analysis

Proteins were separated by SDS-PAGE and transferred to polyvinylidene fluoride (PVDF) membranes. After blocking with 3% BSA in TBS-T (137 mM NaCl, 20 mM Tris-Cl, pH 7.6, and 0.1% Tween 20), membranes were incubated with various primary antibodies, including rabbit polyclonal anti-CRBN (Sigma, Darmstadt, Germany, HPA045910, 1:2000), mouse monoclonal anti-AMPK α (Invitrogen, Waltham, MA, USA, AOH1332, 1:1000), rabbit polyclonal anti-AMPK β (Cell Signaling, Danvers, MA, USA, 4150, 1:1,000), rabbit polyclonal anti-AMPK γ1 (Abcam, Cambridge, UK, ab32508, 1:1000), rabbit polyclonal anti-β-actin (Sigma, A2066, 1:5000), rabbit polyclonal anti-GAPDH (Abfrontier, Seoul, Korea, LF-PA0018, 1:5000), mouse monoclonal anti-Myc (Cell Signaling, 2276, 1:2500), and mouse monoclonal anti-HA (Cell Signaling, 2367, 1:2000). The blots were then incubated with horseradish peroxidase (HRP)-conjugated anti-rabbit or mouse secondary antibody (Jackson ImmunoResearch, West Grove, PA, USA, 111-035-003 or 115-035-008, 1:10,000) and developed using enhanced chemiluminescence detection (ECL; Cytiva, Marlborough, MA, USA).

### 4.4. Co-Immunoprecipitation

Transfected cells were solubilized in radioimmunoprecipitation assay buffer (RIPA buffer: 20 mM HEPES, pH 7.4, 150 mM NaCl, 1 mM EDTA, 1 mM EGTA, 1% Triton X-100, 1% NP-40, 1% sodium deoxycholate, 2 mM Na_3_VO_4_, 100 mM NaF, and 1 mM PMSF) containing a protease inhibitor cocktail. The lysates were centrifuged at 10,000× *g* for 15 min at 4 °C. After centrifugation, the supernatant was incubated with antibodies (anti-AMPK α or anti-Myc antibodies) for 16 h at 4 °C. Antibody–protein complexes were precipitated with protein G beads (GE Healthcare Biosciences), equilibrated in RIPA buffer for 3 h at 4 °C. The beads–antibody-bound protein complexes were washed three times with ice-cold RIPA buffer. After washing, the immune complexes were incubated with lysis buffer at 95 °C for 10 min. The supernatants were subjected to Western blot analysis.

### 4.5. Statistical Analysis

All values represent the mean ± SEM. Significant differences between groups were determined using two-tailed, unpaired Student’s *t*-test, and multiple comparisons were performed using one-way ANOVA or two-way repeated-measures ANOVA. Differences with *p* < 0.05 were considered statistically significant and are shown in the figure legends.
